# Supplementary material for: EqSpike: spike-driven equilibrium propagation for neuromorphic implementations
Source: iScience. 2021 Feb 20;24(3):102222. doi: 10.1016/j.isci.2021.102222 (PMC7970361; doi:10.1016/j.isci.2021.102222)
Supplement: Document S1. Transparent methods and Tables S1 and S2 [file mmc1.pdf]

iScience, Volume *24*

Supplemental information

EqSpike: spike-driven

equilibrium propagation

for neuromorphic implementations

Erwann Martin, Maxence Ernoult, Jérémie Laydevant, Shuai Li, Damien Querlioz, Teodora Petrisor, and Julie Grollier

Supplementary information for:

EqSpike: Spike-driven Equilibrium Propagation for Neuromorphic Implementations

Erwann Martin[1], Maxence Ernoult[2,3], Jérémie Laydevant[2], Shuai Li[2], Damien Querlioz[3], Teodora Petrisor[1], Julie Grollier[2]

[1] - Thales Research and Technology, 91767 Palaiseau, France

[2] - Unité Mixte de Physique, CNRS, Thales, Université Paris-Saclay, 91767 Palaiseau, France

[3] - Université Paris-Saclay, CNRS, Centre de Nanosciences et de Nanotechnologies, 91120 Palaiseau, France

Transparent Methods:

Spiking neurons

We use leaky-integrate-and-fire (LIF) neurons whose membrane potentials follow the standard equation (*34*):

$$\frac{du_i}{dt} = -\gamma_{LIF}\, u_i + I_i(t),$$

where γ_{LIF} is the leak factor and $I_i(t)$ the input signal of neuron i at time t :
$I_i(t) = \sum_{j=1}^{N_{neuron}} W_{ij}\, \delta(t - t_j)$, where δ is the Dirac function and t_j are the times at which neurons j spike.

The neuron integrates input signals until its membrane potential u_i overcomes a threshold value u_{th}. At that moment, the neuron declares a spike, the threshold value u_{th} is subtracted from u_i, and the neuron undergoes a refractory period of duration $T_{refract}$.

Simulation parameters

The values of the different parameters used in simulations are listed in Table 1 of the, where time is expressed in units of $T_{refract}$.

Simulation timestep, dt ($T_{refract}$)	γ_{LIF}	γ_{LI}	u_{th}	τ ($T_{refract}$)	η_r	N_{filt}	β	T_{free} ($T_{refract}$)	T_{nudge} ($T_{refract}$)
0.5	0.01	0.1	1	50	3x10^{-6}	10	0.5	75	100

Table S1. - Simulation parameters, related to all figures and tables in the article.

Algorithm

In the EqSpike algorithm, the rates need to be locally stored for the duration τ, in order to obtain the rate derivative, with τ smaller than the duration of both the free and nudge phases. This is why the neuron rates, ρ, are computed even during the free phase. Indeed, the computation of $\overline{\rho(\dot{t})}$ in the nudging phase requires knowing the values of ρ at $t - \tau$, which may belong to the free phase when t corresponds to the beginning of the nudging phase. In analog hardware, the delay τ would typically be achieved with capacitors comparable to those used for implementing the LIF neurons. In this sense, the EqSpike approach can be considered as local in time as well as in space. Neurons, integrators and filters are initialized to zero at each new image.

Simulations of EqSpike on MNIST

The MNIST dataset contains 60,000 images for training and 10,000 images for test. Pixel values are normalized between 0 and 0.5 before being sent as fixed input signals I to the input neurons. We consider a one-hidden layer bidirectional neural network, with 100 hidden neurons, resulting in the network topology 784-100-10. The loss function that the network attempts to minimize is the mean square error: $L(\hat{y}, y) = \frac{1}{M}\sum_{k=0}^{M}(\widehat{\rho_k} - \rho_k)^2$, where the sum is performed over the output neurons. The target $\widehat{\rho_k}$ corresponds to the maximum neuron frequency f_{max} if the class is correct, and zero otherwise. During the nudging phase, the output neurons integrate the error derivative $(\widehat{\rho_k} - \rho_k)$ multiplied by the nudging factor β in addition to the currents that they receive from other neurons. In practice ρ_k is the rate evaluated over 100 simulation time steps. The batch size is one. Parameters in Table 1 have been optimized through hyperparameter search.

Training parameters for benchmark

Our rate-based benchmark with C-EP and BPTT use the prototypical models introduced in Ernoult et al with rate-based neurons (*33*). The table below describes the hyperparameters used for the simulations.

	Alg	Topology	Activation	T1	T2	beta	Learning rates
MNIST	BPTT	784-100-10	hardsigm	30	15	NA	0.003 – 0.0015
MNIST	C-EP	784-100-10	hardsigm	30	15	0.5	0.003 – 0.0015

Table S2 - Parameters used for benchmarking EqSpike to BPTT and C-EP, related to Table 1.
